# Case Report: Congenital candidemia due to non-*albicans Candida* species–A report of two cases and literature review

**DOI:** 10.3389/fmed.2025.1614725

**Published:** 2025-07-01

**Authors:** Xiaojing Yu, Lan Zhang, Lijun Wen, Yuli Zhong, Minxu Li

**Affiliations:** Department of Neonatology, Dongguan Maternal and Child Health Care Hospital, Dongguan, Guangdong, China

**Keywords:** congenital systemic candidiasis, candidemia, non*-Candida albicans* infection, intrauterine infection, literature review

## Abstract

Congenital candidiasis caused by non-*albicans Candida* (NAC) species represents a life-threatening condition with significant underreporting in clinical literature. It mainly manifests as systemic invasive candidiasis. Premature infants are affected more easily due to the immaturity of their immune system. Risk factors such as those with *in vitro* fertilization (IVF)-associated, premature membrane rupture, and the presence of intrauterine devices are reported to be associated with congenital systemic candidiasis (CSC). We present two cases of extremely preterm neonates diagnosed with congenital systemic candidiasis (CSC): case 1 involved *Candida tropicalis* infection, while case 2 exhibited *Candida glabrata* infection. Based on the literature reports, we conduct a review on CSC due to NAC. We highlight the importance of early recognition and treatment in neonates born to mothers with risk factors. It is recommended that maternal screening for candidiasis and prophylactic antifungal treatment should be conducted promptly for mothers with risk factors when signs of preterm birth appear. CSC should be taken into consideration when unaccountable disseminated infections occur in preterm infants and antifungal therapy should be administered as soon as possible.

## Introduction

Congenital candidiasis is a severe but rare complication of candidal vulvovaginitis. It is classified into in two forms: congenital cutaneous candidiasis (CCC) characterized by diffuse erythematous papulopustular eruptions, and CSC involving multi-organ dissemination. Although candidal vulvovaginitis occurs 10%−35% of pregnancy, but < 1% of candidal vulvovaginitis develop candidial chorioamnionitis with fetus involvement (1). Vertical transmission via ascending infection, predominantly involving *Candida* species colonizing the lower genital tract, induces chorioamnionitis, subsequently leading to disseminated neonatal candidiasis. The most important maternal risk factors associated with CSC is the presence of a foreign body in the uterus, such as an contraceptive device or cervical suture. Morever, extensive instrumentation in the delivery room and invasive procedures in the newborn may increase the risk for subsequent development of CSC (2). According to the literature, CCC only occurred in 0.1% of all neonatal intensive care units admissions (3), while non-*albicans Candida* (NAC)-associated CSC are more seldom seen. A comprehensive meta-analysis developed in 2020 found that only 44 recognized cases had been reported over the past 54 years (1, 2). Here, we report two extremely preterm neonates with NAC-associated CSC: case 1 exhibited *Candida tropicalis* sepsis with multi-organ involvement, while case 2 presented *Candida glabrata* sepsis with pulmonary and gastrointestinal tract involvement. A systematic review of 17 NAC-associated CSC cases was conducted in this study.

## Case report

### Case 1

**Figure 1:**
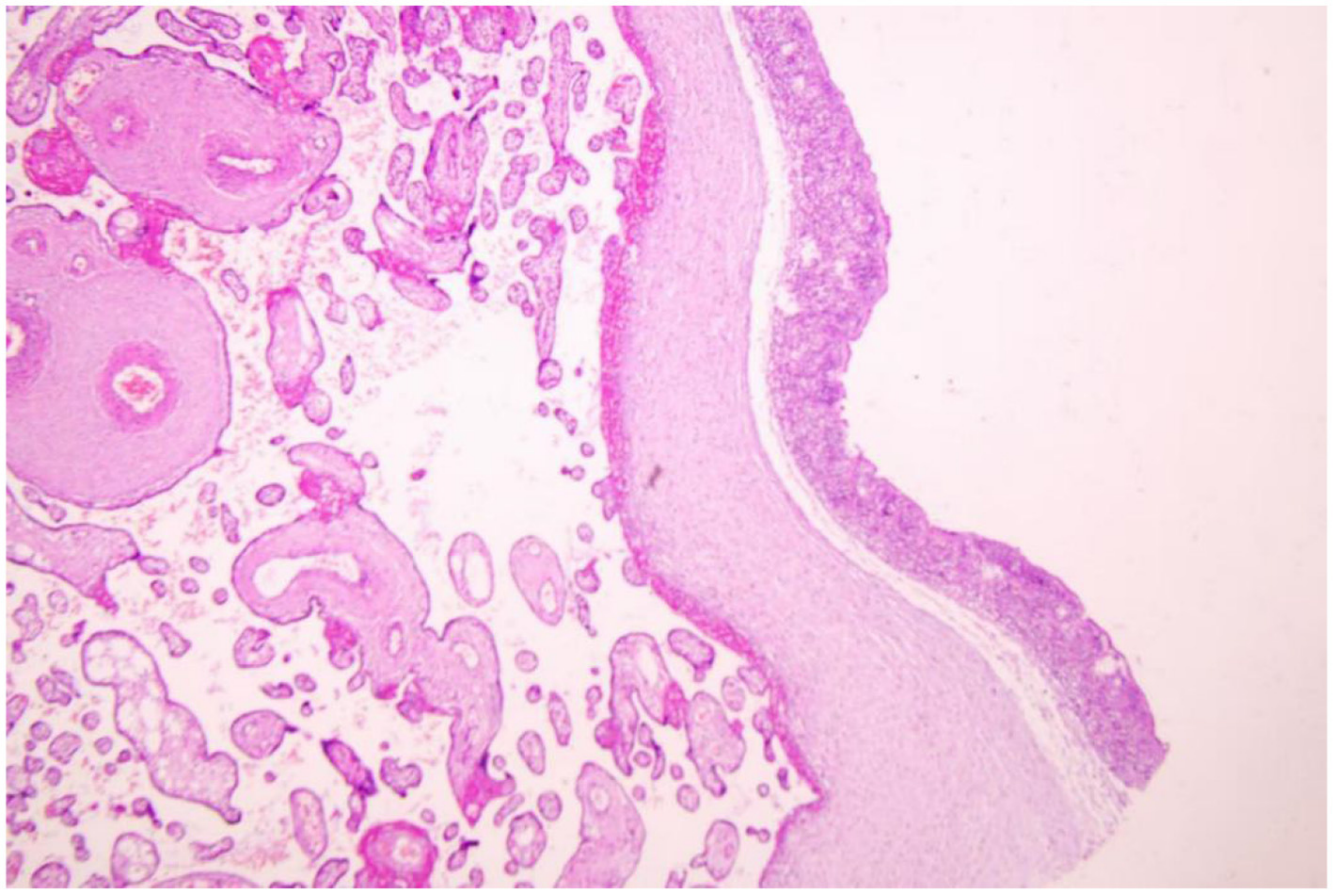
Histopathology photograph of candidal chorioamnionitis.

A 27-year-old woman, gravida 2, para 1, was admitted to our hospital at 27^+1^ weeks of gestation with signs of preterm labor and cervical effacement. She was conceived by *in vitro* fertilization (IVF). There was no remarkable history during pregnancy, including *Candida* vaginitis. Blood examination showed white blood cell count of 18.85 × 10^9^/L and C-reactive protein (CRP) level of 87.58 mg/L; blood cultures were negative after 72 h. Magnesium sulfate tocolysis and dexamethasone therapy was administered. However, premature rupture of the membranes occurred at 27^+3^ weeks of gestation. On day 3 postpartum, the culture of her vaginal discharge, collected on admission, identified *C. tropicalis*. She was treated with a single oral dose of Fluconazole (150 mg), and she responded well to the medication. Histopathological examination of the placenta and umbilical cord revealed acute chorioamnionitis and umbilical vasculitis (Figure 1).

A living male extremely premature infant weighing 1.27 kg was delivered vaginally at 27^+3^ weeks of gestation. His Apgar scores was 10, 10, and 10 at 1, 5, and 10 min. He was referred to Newborn Intensive Care Unit (NICU) because of severe respiratory distress and extremely premature. Chest radiographs showed white lungs and High Frequency Oscillatory Ventilation was initiated due to the high oxygen requirements and progressive respiratory distress. Endotracheal surfactant therapy was also promptly administered. Blood examination showed white blood cell count of 9.97 × 10^9^/L, platelet count of 82 × 10^9^/L, and CRP level of 36.13 mg/L. Empiric antibiotic therapy with cefotaxime and ampicillin was initiated intravenously. At 20 h of life, he developed high fever. On day 3 of life, he became pale and developed bruising. Both blood cultures taken from the umbilical arterial and peripheral grew *C. tropicalis*, which was sensitive to amphotericin B and fluconazole. Base on histopathological findings, blood culture and clinical manifestations, congenital candidiasis due to *C. tropicalis* was diagnosed and intravenous fluconazole (6 mg/kg/day) was administered. Cerebrospinal fluid culture was negative. Although antifungal therapy and supportive treatments were administered promptly, his clinical condition became deteriorated rapidly. On day 10, signs of septic shock, including poorly purse, hypotension, systemic edema, oliguria, and hematuria, occurred and high fever still persisted. Additionally, recurrent hypoglycemia was noted and was difficult to correct. Repeated blood cultures were constantly positive for *C. tropicalis*. Sputum and ascitic fluid cultures also revealed the presence of *C. tropicalis*. Repeated blood examinations revealed progressive increase in white blood cell count to 51.88 × 10^9^/L and decrease in platelet count to 8 × 10^9^/L. Infection remained uncontrolled, combination therapy with amphotericin B (1 mg/kg/day) and fluconazole (6 mg/kg/day) was started intravenously. Terminal complications included grade IV intraventricular hemorrhage, liver dysfunction, and acute renal failure. On day 19, he died and blood cultures continued to grow *C. tropicalis*.

### Case 2

A 30-year-old woman, gravida 2, para 1, was admitted to our hospital at 26 weeks of gestation due to vaginal bleeding and uterine contractions. She conceived spontaneously, and the pregnancy course had been uneventful. Her vital signs were stable on admission. The blood examination revealed a white blood cell count of 10.8 × 10^9^/L and a CRP level of 2.4 mg/L. Ritodrine hydrochloride and magnesium sulfate tocolysis were administered intravenously regularly. Dexamethasone therapy was also administered to enhance fetal lung maturity. However, uterine contractions were not controlled. She was diagnosed with threatened premature delivery and she terminated the gestation by cesarean section at 26^+1^ weeks of gestation. The maternal vaginal culture obtained upon admission yielded *C. glabrata* on postpartum day 3. She did not receive treatment for vaginal candidiasis prior to delivery. Following delivery, she was treated with the local administration of nystatin and ultimately recovered. Histopathological examination of the placenta revealed infiltration of neutrophils without chorioamnionitis.

The living male infant, weighing 0.89 kg, had Apgar scores of 2, 7, and 9 at 1, 5, and 10 min, respectively. After cardiopulmonary resuscitation, his vital signs stabilized, and he was admitted to NICU. Chest radiographs indicated hyaline membrane disease (III°). Blood examination showed white blood cell count of 8.26 × 10^9^/L, platelet count of 93 × 10^9^/L, and CRP level of 0.8 mg/L. Initial therapy included conventional mechanical ventilation, administration of endotracheal surfactant, parenteral nutrition, and empiric antibiotic therapy (cefotaxime and ampicillin). On day 2 of life, enteral feeding with breast milk was initiated. On day 4 of life, he developed fever and abdominal distention. Blood cultures were repeated since the blood cultures taken from the umbilical arterial and peripheral were sterile. In addition, metagenomic next-generation sequencing (mNGS) of serum was detected to identify infectious agents. The examination of cerebrospinal fluid ruled out intracranial infection. Neonatal Necrotizing Enterocolitis (NEC) was suspected and the antibiotic therapy was replaced with injection piperacillin/tazobacta. On day 6 of life, abdominal plain film revealed acute intestinal perforation. Meanwhile, mNGS detected *C. glabrata*. A diagnosis of CSC due to *C. glabrata* was subsequently established. Intravenous fluconazole (6 mg/kg/day) was administered immediately. His clinical condition deteriorated, with the development of massive ascites and hypotension. Repeat blood examination showed white blood cell count of 17.67 × 10^9^/L, platelet count of 75 × 10^9^/L, and CRP level of 24.72 mg/L. Intravenous fluconazole was administered immediately. The clinical condition was worsen for the appearance of massive ascites and hypotension. Recheck blood examination showed white blood cell count of 17.67 × 10^9/^L, platelet count of 75 × 10^9/^L, and CRP level of 24.72 mg/L. On day 7 of life, he underwent an urgent surgery to resect the necrotic distal ileum. Culture of ascitic fluid yielded *C. glabrata* finally. Antifungal treatment lasted for 6 weeks. He was discharged after hospitalization for 68 days.

## Methods

A comprehensive search was conducted using the keywords “congenital systemic candidiasis,” “congenital candidemia,” “neonatal fungal sepsis,” “non-*Candida albicans* infection,” and “intrauterine *Candida* infection” in the PubMed, China National Knowledge Infrastructure, and Wanfang databases to identify all relevant papers published between 1966 and 2024. This is a retrospectively study. CSC presents as systemic infection, with positive blood, urine, and/or cerebrospinal fluid cultures for *Candida* spp. The demonstration of *Candida* in histopathological and culture specimens obtained at autopsy is also regarded as CSC. Only cases diagnosed with CSC caused by NAC species have been included in our review. CSC cases caused by *C. albicans* and hospital-acquired NAC infections are excluded. Relevant cases were reviewed independently by all of the authors. The eligibility of each case were assessed with standardized data abstraction forms.

## Result

In addition to our two cases, 17 cases of CSC caused by NAC, including two sets of twins, have been identified in total. The clinical features of the mothers and the infants were summarized in Tables 1, 2, respectively. Since some information is not mentioned in the original paper, we have compiled the certain data. Seven mothers (7/17, 41.2%) were conceived by IVF. Seven mothers (7/17, 41.2%) had a history of premature membrane rupture. Twelve mothers (12/17, 70.6%) experienced vaginal infection, yet most did not receive antifungal treatment during pregnancy. Five of 19 infants (26.3%) died. *C. glabrata* is the most common agent, accounting 8 cases, followed by *C. tropicalis* (3 cases), *C. krusei* (3 cases), *C. parapsilosis* (2 cases), *C. kefyr* (2 case), *C. ciferri* (1 case). Of the infants, 89.5% (17/19) were premature.

**Table 1:** Clinical features of mothers during pregnancy.

**Cases**	**Conceived with IVF**	**Maternal infection**	**Prebirth antifungal treatment**	**Premature rupture of membranes**	**Mode of delivery**	**GA**	**Histopathological discovery**
1 (17)	NA	NA	NA	NA	C-section	Term	NA
2 (20)	NA	Amniotic fluid culture showed *C. glabrata*	Fluconazole intravenously for 2 weeks	No	C-section	29 weeks	Mild chorioamnionitis
3 (11)	No	*C. tropicalis* vaginitis	No	Yes	Vb	31 weeks	Focal acute funisitis and acute chorioamnionitis including budding yeasts and hyphae
4 (7)	Yes	NA	No	NA	Vb	26^+4^ weeks	NA
5 (21)	NA	Tuberculosis	NA	NA	NA	28 weeks	NA
6 (22)	NA	Chorioamnionitis	No	Yes	Vb	25 weeks	NA
7 (23)	NA	*C. ciferri* vaginitis	NA	No	Vb	Term	NA
8 (24)	Yes	Chorioamnionitis	No	Yes	C-section	29 weeks	Acute chorioamnionitis and deciduitis
9 (25)	NA	Chorioamnionitis	No	Yes	Vb	25 weeks	NA
10 (25)	NA	No	No	Yes	C-section	31 weeks	NA
11 (26)	Yes	Candidal vaginitis	NA	NA	Vb	30 weeks	NA
12 (4)	Yes	*C. glabrata* vaginitis	Topical therapy	NA	C-section	26 weeks	Stage 3 acute chorioamnionitis; Microscopically, severe subacute funisitis, with diffuse necrosis of the amnion, was revealed
13 (4)	NA	NA	NA	NA	C-section	25 weeks	Stage 3 acute chorioamnionitis; The umbilical cord revealed microscopically necrotizing funisitis
14 (27)	Yes	Recurrent yeast vaginitis	No	No	C-section	34 weeks	Numerous foci of budding yeasts were demonstrated on membranes and the yeasts were compatible in size with *C. glabrata* species.
15 (28)	Yes	Candidal vaginitis	No	Yes	C-section	25 weeks	Acute *C. glabrata* chorioamnionitis.
16 (case 1)	Yes	*C. tropicalis* vaginitis	No	Yes	Vb	27^+3^ weeks	Acute chorioamnionitis and umbilical vasculitis
17 (case 2)	No	*C. glabrata* vaginitis	No	No	C-section	26^+1^ weeks	Infiltration of neutrophils without chorioamnionitis

C-sention, cesarean section; Vb, vaginal birth.

**Table 2:** Clinical features of infants with congenital systemic candidiasis.

**Cases**	**Sex**	**Infectious agent**	**Postive culture/HP**	**Blood examination**	**Presentation**	**Antifungal therapy**	**Outcome**
1 (17)	M	*C. tropicalis*	Blood	Low platelets	Rash, icterus kept on worsening firm hepatomegaly, infective endocarditis and gall bladder masses	AmB for 2 weeks, replaced with AmBisome and FCZ for 6 weeks	Survive
2 (20)	M	*C. glabrata*	Skin, throat, sputum and blood	Normal	Septic shock, pulmonary hemorrhage and grade IV intraventricular hemorrhage	FCZ	Survive
3 (11)	M	*C. tropicalis*	Blood, cerebrospinal fluid, and tracheal aspirate, HP	Low platelets	Hypotension, invasive fungal infection	AmB	Dead
4 (7)	M	*C. parapsilosis*	Blood	NA	Distress syndrome	AmB	Dead
5 (21)	M	*C. krusei*	Blood, gastric lavage	Normal	Respiratory distress syndrome	AmB	Survive
6 (22)	M	*C. parapsilosis* and *C. albicans*	Necrosis of the skin and subcuta-neous tissue, blood and tracheal aspirate specimens	Increasing WBC	truNACl ec-chymosis with central necrosis, necrotic papule, necrotizing enterocolitis	AmB for 5 weeks	Survive
7 (23)	M	*C. ciferri*	Blood, urine	Normal	Lethargic, feeble cry, multiple tiny vesicles with erythema over face, trunk, and extremities	Intravenous AmB and topical miconazole for 14 days	Survive
8 (24) (Twin B)	M	*C. kefyr*	Blood, HP	NA	Respiratory distress	AmBisome for 3 weeks	Survive
9 (24) (Twin A)	M	*C. kefyr*	HP	Leukopenia	Respiratory distress	AmBisome for 2 weeks	Survive
10 (25)	F	*C. krusei*.	Blood	NA	Hypothermic, hyperglycaemic, intraventri cular hemorrhage with intracranial hypertension	FCZ for 11 days, replaced with AmBisome later	Survive
11 (25)	M	*C. krusei*.	Blood	NA	Sepsis, Ileal perforation	AmBisome for 5 days and FCZ for 19 days	Survive
12 (26)	M	*C. glabrata*	Blood, sputum	Increasing WBC, low platelets	Pneumonia, NEC	AmBisome	Survive
13 (4)	NA	*C. glabrata*	Nasopharynx, the chorionic plate	NA	Necrotizing pneumonia	No	Dead
14 (4)	M	*C. glabrata*	The placenta and the amnion, lung tissue at autopsy	NA	Pneumonia complicated by persistent pulmonary hypertension	No	Dead
15 (27) (Twin A)	M	*C. glabrata*	Blood, gastric fluids, HP	NA	Apnea	Triflucan	Survive
16 (27) (Twin B)	F	*C. glabrata*	Blood, gastric fluids, HP	NA	NA	Triflucan	Survive
17 (28)	NA	*C. glabrata*	Gastric aspirate, sub-phrenic collection, HP	NA	Neonatal peritonitis	FCZ, replaced with 5-FC band FCZ, total duration 43 days	Survive
18 (case 1)	M	*C. tropicalis*	Blood, sputum, ascitic fluid	Leukemoid reaction, low platelets	Respiratory distress, septic shock, intraventricular hemorrhage, liver dysfunction and renal failure	FCZ for 5 days, replaced with AmB band FCZ for 11 days	Dead
19 (case 2)	M	*C. glabrata*	Blood, ascitic fluid	Increasing WBC, low platelets	Respiratory distress, Ileal perforation	FCZ for 6 weeks	Survive

M, male; F, female; GA, gestational age in weeks; NA, not vailable; HP, histopathological; FCZ, fluconazole; AmB, amphotericin B; AmBisome, Liposomal amphotericin B; 5-FC, flucytosine.

## Discussion

Unlike nosocomial systemic candidiasis, CSC is attributed to ascending intrauterine infection with *Candida* spp. Until 2019, there have been < 50 cases of CSC reported in the literature (2), and the number of reported cases appear to increase in recent years. In previous reports, *C. albicans* was the predominant pathogen associated with CSC, whereas cases caused by NAC were rare. Herein, we describe two cases of CSC caused by *C. tropicalis* and *C. glabrata*. Generally, NAC is known to form blastospores without pseudohyphae, which leads to weak virulence and tissue invasion. This may be responsible for the rarity of CSC compared to the *C. albicans*. Yellow-white nodules on the umbilical cord are the characteristic of congenital *Candida* infections and are typically observed in cases involving *C. albicans* due to its invasion of pseudohyphae. Both of our current cases lacked macroscopic nodules on the placentas and umbilical cords. Matsuzawa et al. (4) reported that two extremely premature infants (cases 13 and 14) died due to necrotizing pneumonia caused by *C. glabrata*, which also lacked such macroscopic nodules. Therefore, they suggested that clinicians should not rule out *Candida* in the absence of yellow-white nodules.

The exact mechanisms by which *Candida* spp ascend to the upper genital tract and cause chorioamnionitis remain unknown. To our knowledge, several cases had demonstrated that chorioamnionitis associated with NAC led to stillborns (5, 6). Krallis et al. (7) described cases of congenital candidiasis caused by *C. albicans* and *C. parapsilosis* in extremely premature twins who died within 72 h after birth. Reported risk factors include IVF-associated, premature membrane rupture, the presence of intrauterine devices, maternal history of cervical cerclage, amniocentesis and prenatal exposure to Corticosteroids (2, 8, 9). In our review, we observe that more than 40% mothers have the history of IVF and premature membrane rupture. In the present case 1, CSC was likely linked to the transmission of acute asymptomatic *C. tropicalis* vaginitis, rather the result of IVF. *C. glabrata* is the predominant NAC in candidal candidiasis, with a prevalence of 9% (10). Similarly, *C. glabrata* is also the most common pathogen in CSC in our review. Candidal candidiasis may result in preterm birth, which may account for the phenomenon that most cases in our review are premature. Prophylactic antimycotic treatment is recommended in cases with asymptomatic colonization during the last 6 weeks during pregnancy to prevent transmission to the newborn (10). Unfortunately, prophylactic antimycotic treatment was not administered to both mothers in our cases leading to perinatal transmission of infection in newborns. Therefore, we suggest that maternal screening for candidiasis should be conducted and prophylactic antifungal treatments should be administered positively for these with risk factors when signs of preterm birth occur.

The most frequent clinical manifestations include septic, respiratory distress, and gastrointestinal tract infection. Unlike CCC, skin lessons were not a common feature in our review, with only two cases were conformed to have skin involvement. At least five cases presented with thrombocytopenia and fluctuating white blood cell counts. Leukemoid reactions and thrombocytopenia were also documented in other reported cases with CSC (9, 11–13). Persistent hyperglycemia has been considered to be a significant feature of systemic candidiasis (14), however, on the contrary, ongoing hypoglycemia was observed in case 1. The recurrent, unexplained hypoglycemia, persistent positive blood cultures, and irreversible thrombocytopenia observed in case 1 might indicate a fulminant course and severe infection. Usually, the fungus forms biofilms on biotic and abiotic surfaces, enhancing its resistance to antifungal agents and immune responses. Study revealed that *C. tropicalis* is significantly easier to form a biofilm than other fungus in cases of bloodstream infections (15). We suspect that *C. tropicalis* in case 1 formed biofilms on the infected organ and the surface of the endotracheal intubation, which led to the persistent positive blood cultures. When clinical manifestations are disseminated in premature infants born to mothers with risk factors, CSC should be considered.

Literature review revealed that mortality of untreated systemic candidiasis ranges from 39 to 94% (2). In our review, five of 19 infants (26.3%) died, two of whom had not received antifungal treatments. The cause of death was demonstrated by the autopsy finally. Risk factors contributing to mortality include prematurity, low birth weight neonates, and extensive cutaneous involvement (2). Since CSC often progresses rapidly, even prompt initiation of antifungal therapy may sometimes be ineffective. Clinical Practice Guideline recommends that early and prolonged intravenous antifungal therapy reduces the dissemination and mortality associated with candidiasis. Amphotericin B and fluconazole are recommended as the first-line treatment of congenital candidiasis (16). In 2012, Jajoo et al. (17) reported a case of *C. tropicalis* infection in a term neonate presenting with gallbladder masses and infective endocarditis, which failed conventional amphotericin B therapy but was successfully treated with liposomal amphotericin B and fluconazole. Unfortunately, neither the intravenous administration of fluconazole nor the combination of amphotericin B with fluconazole showed improvement for the persistent candidemia or prevented further clinical deterioration in our case 1. In recent years, antifungal resistance rates have been increasing. Recent findings suggested that the mechanisms of azole resistance in *C. tropicalis* are related to mutations and overexpression in drug target genes such as ERG11 (18). Changes in the cell wall and membrane of *C. tropicalis* may result in resistance to amphotericin B (19). The reason why *C. tropicalis* in case 1 is resistant to amphotericin B and fluconazole remains unknown. A prospective study suggested that empiric antifungal treatment should be considered for extremely low birth weigh infants delivered vaginally who present with pneumonia and whose mothers have chorioamnionitis or an intrauterine foreign body (9).

## Conclusion

In conclusion, we highlight the significance of early recognition and treatment for CSC. It is recommended that maternal screening for candidiasis and prophylactic antifungal treatment should be conducted promptly for mothers with risk factors when signs of preterm birth appear. CSC should be taken into consideration when unaccountable disseminated infections occur in preterm infants and antifungal therapy should be administered as soon as possible.

## Data Availability

The raw data supporting the conclusions of this article will be made available by the authors, without undue reservation.
